# Bevacizumab treatment of meningeal melanoma metastases

**DOI:** 10.1186/s12967-020-02212-3

**Published:** 2020-01-08

**Authors:** Trude G. Simonsen, Jon-Vidar Gaustad, Einar K. Rofstad

**Affiliations:** Group of Radiation Biology and Tumor Physiology, Department of Radiation Biology, Institute for Cancer Research, Oslo University Hospital, Oslo, Norway

**Keywords:** Melanoma, Meningeal metastasis, Antiangiogenic therapy, Bevacizumab, VEGF

## Abstract

**Background:**

Melanoma patients with metastatic growth in the meninges have poor prognosis and few treatment options. Although treatment with BRAF inhibitors or immune checkpoint inhibitors has provided promising results, most patients with advanced melanoma are resistant to these treatments and develop severe side effects. Novel treatment strategies are needed for patients with meningeal melanoma metastases, and the potential of antiangiogenic therapy was investigated in this preclinical study.

**Methods:**

Two GFP-transfected melanoma models (A-07 and D-12) differing substantially in VEGF-A expression were included in the study, and the anti-VEGF-A antibody bevacizumab was used as therapeutic agent. Meningeal metastases were initiated in BALB/c *nu*/*nu* mice by intracranial inoculation of melanoma cells, and bevacizumab treatment was given twice a week in i.p. doses of 10 mg/kg until the mice became moribund. Therapeutic effects were evaluated by determining tumor host survival time, assessing tumor growth and angiogenic activity by quantitative analyses of histological preparations, and measuring the expression of angiogenesis-related genes by quantitative PCR.

**Results:**

Meningeal A-07 melanomas showed higher expression of VEGF-A than meningeal D-12 melanomas, whereas the expression of ANGPT2 and IL8, two important angiogenesis drivers in melanoma, was much higher in D-12 than in A-07 tumors. Bevacizumab treatment inhibited tumor angiogenesis and prolonged host survival in mice with A-07 tumors but not in mice with D-12 tumors. Meningeal A-07 tumors in bevacizumab-treated mice compensated for the reduced VEGF-A activity by up-regulating a large number of angiogenesis-related genes, including ANGPT2 and its receptors TIE1 and TIE2. Melanoma cells migrated from meningeal tumors into the cerebrum, where they initiated metastatic growth by vessel co-option. In the A-07 model, the density of cerebral micrometastases was higher in bevacizumab-treated than in untreated mice, either because bevacizumab treatment increased mouse survival or induced increased tumor gene expression.

**Conclusions:**

The development of antiangiogenic strategies for the treatment of meningeal melanoma metastases is a challenging task because the outcome of treatment will depend on the angiogenic signature of the tumor tissue, treatment-induced alterations of the angiogenic signature, and the treatment sensitivity of metastatic lesions in other intracranial sites.

## Background

Patients diagnosed with malignant melanoma have a high risk of developing brain metastases [1]. Approximately 50% of the patients with advanced-stage disease show brain involvement during the course of their disease, and this incidence has increased to ~ 75% at autopsy [2–4]. The prognosis of the patients with brain involvement is poor, with a median overall survival of 4–6 months [2, 5, 6]. A majority of these patients show multiple brain lesions and/or meningeal involvement, and these factors are associated with particularly poor prognosis [2, 5]. While current treatment options for patients with a limited number of brain parenchymal lesions include surgical resection and stereotactic radiosurgery, the treatment options for patients with multifocal disease and meningeal involvement are mainly palliative and include whole-brain radiotherapy and chemotherapy [1, 7, 8]. Recently, the introduction of molecularly targeted therapies and immunotherapy has given hope to patients with advanced melanoma [9, 10], and several trials are evaluating the effect of BRAF inhibitors and immune checkpoint inhibitors in patients with brain metastases [1, 11]. Although initial results are encouraging, a substantial proportion of the patients show intrinsic or acquired treatment resistance, and the drugs are also associated with severe side effects [9, 11]. Consequently, it is important to explore further treatment strategies to improve the dismal prognosis of this patient group.

The high incidence of brain involvement in autopsy studies suggests that subclinical brain lesions are common in melanoma patients [3, 12]. With prolonged patient survival due to improved control of extracranial disease, these subclinical lesions may be allowed more time to develop and grow into symptomatic brain metastases [13]. Brain metastases cause deteriorating and sometimes irreversible neurological symptoms, and a strategy to prevent the growth of brain metastases would be of great clinical benefit [13]. It is well established that melanoma growth and progression depend on angiogenesis, and vascular endothelial growth factor A (VEGF-A) has been shown to be an important angiogenic driver in melanoma [14]. Antiangiogenic therapy targeting the VEGF-A pathway has thus been suggested as a possible strategy to prevent melanoma relapse and spread after locoregional surgery, and adjuvant treatment with the anti-VEGF-A antibody bevacizumab [15] is currently being investigated in a large multicenter randomized phase 3 trial in melanoma patients with high risk of recurrence [16]. Furthermore, high expression of VEGF-A has been associated with the vascularization and growth of melanoma brain metastases in preclinical studies [17–20], and a recent study including both clinical and preclinical experiments suggests that bevacizumab can prevent the formation of brain metastases in lung adenocarcinoma [21]. Antiangiogenic therapy targeting the VEGF-A pathway may thus represent a potential strategy to prevent metastasis to the brain in melanoma patients.

Therapeutic effects of antiangiogenic agents in melanoma brain metastases have been examined in a few preclinical studies [22–24], and these studies have provided varying results, possibly reflecting a complex role of angiogenesis and VEGF-A in the formation and growth of metastases in the brain [1]. The vascularization of melanoma brain metastases may occur by sprouting angiogenesis (i.e., the sprouting of new vessels from pre-existing vessels) as well as vessel co-option (i.e., the growth of tumor cells along pre-existing vessels without the formation of new vessels) [19, 23, 25, 26]. Although high VEGF-A expression is generally associated with sprouting angiogenesis [19], vessel co-option has also been observed in melanoma models with high VEGF-A expression [27], and VEGF-A has been shown to induce progression of experimental melanoma brain metastases without inducing sprouting angiogenesis [17]. Moreover, different VEGF-A isoforms may have differential effects on the development and growth of metastatic disease in the brain [18].

Melanomas may form metastases in multiple intracranial sites, and these metastases may differ in vascularization and response to antiangiogenic agents [23, 24, 27]. Meningeal metastases show signal enhancement in contrast-enhanced MRI, suggesting leaky vessels and sprouting angiogenesis [8]. Furthermore, the level of VEGF-A in the cerebrospinal fluid has been shown to have diagnostic and prognostic value [28]. These observations suggest that meningeal lesions may be susceptible to antiangiogenic agents targeting the VEGF-A pathway. This possibility was investigated in the study reported in the present communication. Two preclinical models of meningeal melanoma metastases differing highly in VEGF-A expression were included in the study, and bevacizumab was used as therapeutic agent. Effects of treatment were evaluated by assessing tumor host survival, analyzing tumor growth and vascularity in histological preparations, and measuring the expression of angiogenesis-related genes.

## Materials and methods

### Mice

Adult (8–10 weeks of age) female BALB/c *nu*/*nu* mice were used as host animals. The mice were bred at our institute and maintained under specific pathogen-free conditions at a temperature of 22–24 °C and a humidity of 30–50%. The animal experiments were approved by the Institutional Committee on Research Animal Care, Department of Comparative Medicine, Oslo University Hospital, Norway and the Norwegian Food Safety Authority, Brumunddal, Norway (Approval: FOTS ID 10422), and were performed in accordance with the Interdisciplinary Principles and Guidelines for the Use of Animals in Research, Marketing, and Education (New York Academy of Sciences, New York, NY) and the EU Directive 2010/63/EU for animal experiments (http://ec.europa.eu/environment/chemicals/lab_animals/legislation_en.htm).

### Cell lines

A-07 and D-12 human melanoma cells [29] constitutively transfected with green fluorescence protein (GFP) were obtained from our frozen stock and maintained as monolayers in RPMI 1640 (25 mM HEPES and l-glutamine) medium supplemented with 13% bovine calf serum, 250 µg/mL penicillin, 50 µg/mL streptomycin, and 700 µg/mL (A-07) or 900 µg/mL (D-12) genetecin. The cultures were incubated at 37 °C in a humidified atmosphere of 5% CO_2_ in air and subcultured twice a week. Cells were harvested from exponentially growing cultures and resuspended in Ca^2+^-free and Mg^2+^-free Hanks’ balanced salt solution (HBSS) before injection into animals.

### Anesthesia

Intracranial injection of tumor cells was carried out with anesthetized mice. Fentanyl citrate (Janssen Pharmaceutica, Beerse, Belgium), fluanisone (Janssen Pharmaceutica), and midazolam (Hoffmann-La Roche, Basel, Switzerland) were administered intraperitoneally (i.p.) in doses of 0.63 mg/kg, 20 mg/kg, and 10 mg/kg, respectively. The body core temperature of the mice was maintained at 37–38 °C by using a heating pad.

### Intracranial tumor cell injection

The mice were fixed in a stereotactic apparatus (Model 900; Kopf Instruments, Tujunga, CA) for injection of tumor cells into the right hemisphere. The injection point was 2 mm anterior to the coronal and 1 mm lateral to the sagittal suture lines. A 100-µL Hamilton syringe with a 26-gauge needle was used to inject 3.0 × 10^3^ cells suspended in 6 µL of HBSS. To minimize cell reflux, the cells were injected slowly and the needle was left in place for 2 min before it was retracted slowly.

### Bevacizumab treatment

Bevacizumab (Avastin; Hoffman-La Roche, Basel, Switzerland) was dissolved in physiological saline and administered i.p. in doses of 10 mg/kg. Treatment with bevacizumab or vehicle was initiated 1 day before tumor cell injection, and continued twice a week until the mice became moribund. The mice were examined daily for clinical signs of tumor growth. Moribund mice were killed and autopsied, and the brain was removed for subsequent histological analysis or quantitative PCR of the tumor tissue. The survival time of the mice was calculated as the time from tumor cell injection until the mice became moribund and were euthanized.

### Histological analysis

The brain was fixed in phosphate-buffered 4% paraformaldehyde and embedded in paraffin. Histological sections were cut and stained with hematoxylin and eosin (HE) using standard procedures or immunostained by using a peroxidase-based indirect staining method [30]. An anti-GFP rabbit polyclonal antibody (Abcam, Cambridge, UK) or an anti-CD31 rabbit polyclonal antibody (Abcam) was used as primary antibody to detect melanoma cells or endothelial cells, respectively. Diaminobenzidine was used as chromogen, and counterstaining was carried out with hematoxylin. Microvascular density (MVD) was scored by counting CD31-positive vessels, and tumor angiogenic activity (i.e., the rate of generation of blood vessels) was calculated from the tumor cross-section, MVD, and the time from initiation of angiogenesis to tumor removal [31]. Micrometastases within the cerebral parenchyma were scored by counting colonies of GFP-positive tumor cells. Three brain sections from each mouse were subjected to quantitative analysis.

### Quantitative PCR

The RT^2^ Profiler PCR Array Human Angiogenesis (PAHS-024Z; SABiosciences, Frederick, MD) was used for expression profiling of angiogenesis-related genes. Total RNA was isolated from cultured cells in exponential growth or from tumor tissue stabilized in RNA*later* RNA Stabilization Reagent (Qiagen, Hilden, Germany). RNA isolation, cDNA synthesis, and real-time PCR were performed as described in detail previously [32]. Fold difference in gene expression was calculated by using the ΔΔC_T_-method [33]. A C_T_-value of 35 (15 cycles above the positive PCR control) was set as detection limit. The array included 5 housekeeping genes [β-actin (ACTB), β-2-microglobulin (B2M), glyceraldehyde-3-phosphate dehydrogenase (GAPDH), hypoxanthine phosphoribosyltransferase-1 (HPRT1), and ribosomal protein lateral stalk subunit P0 (RPLP0)], and each C_T_-value was normalized to the mean C_T_-value of these genes as: ΔC_T_ = C_T_^gene of interest^ − C_T_^mean of housekeeping genes^. Normalized gene expression levels were calculated from three biological replicates of cultured cells or from three tumors as 2^−mean ΔCT^.

### Statistical analysis

The Spearman rank order test was used to search for correlations between parameters. Comparisons of survival curves were performed using the log-rank test. Comparisons of other data sets were conducted using the Student’s *t* test when the data complied with the conditions of normality and equal variance. Under other conditions, comparisons were carried out by non-parametric analysis using the Mann–Whitney rank sum test. Probability values of *P* < 0.05 were considered significant. Statistical analysis was performed using the SigmaStat statistical software (SPSS, Chicago, IL).

## Results

### Bevacizumab treatment prolonged the survival of mice with meningeal A-07 tumors

**Fig. 1:**
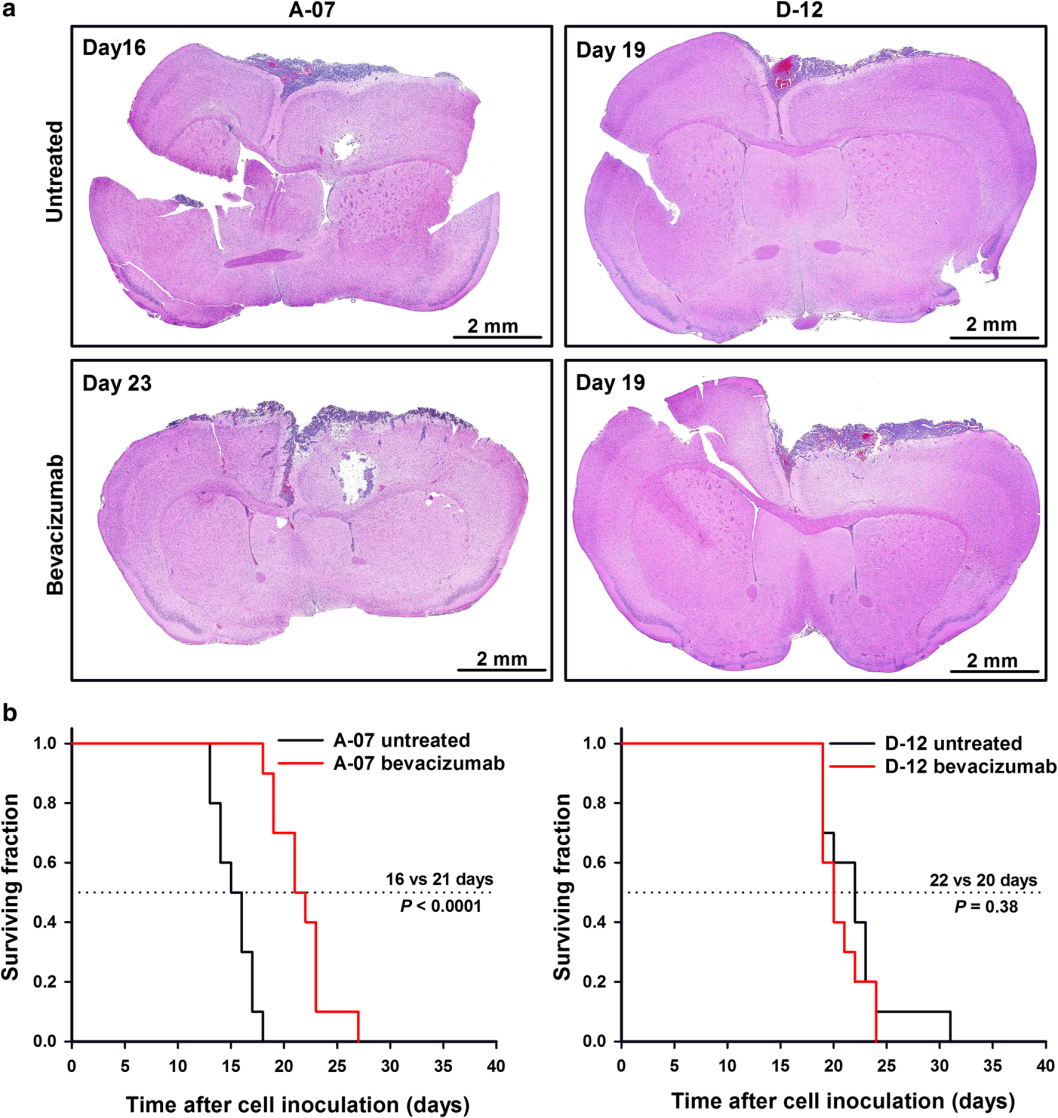
Tumor growth and host survival. **a** Histological preparations of brains from untreated and bevacizumab-treated mice showing that A-07 and D-12 cells developed meningeal tumors after intracranial cell inoculation. The day numbers indicate time after cell inoculation. **b** Fraction of surviving mice versus time after cell inoculation for untreated and bevacizumab-treated mice with meningeal A-07 or D-12 tumors (*N* = 10 in each group). Bevacizumab treatment prolonged the survival of mice with A-07 tumors but not the survival of mice with D-12 tumors. *P*-values: log-rank test

Both melanoma models developed meningeal tumors after intracranial tumor cell inoculation (Fig. 1a). The meningeal tumors induced severe clinical symptoms and limited the survival of the host mice. The most frequent symptoms were weight loss, disconnection of scull sutures, destruction of scull bone, and extracranial bleedings. The clinical symptoms and tumor growth pattern did not differ between bevacizumab-treated and untreated mice in any of the models. The median survival of untreated mice was 16 days (A-07) and 22 days (D-12). Bevacizumab treatment prolonged the median survival of mice with A-07 tumors to 21 days (*P* < 0.0001; Fig. 1b), but did not prolong the median survival of mice with D-12 tumors (*P* = 0.38; Fig. 1b).

### Bevacizumab treatment inhibited angiogenesis in meningeal A-07 tumors

**Fig. 2:**
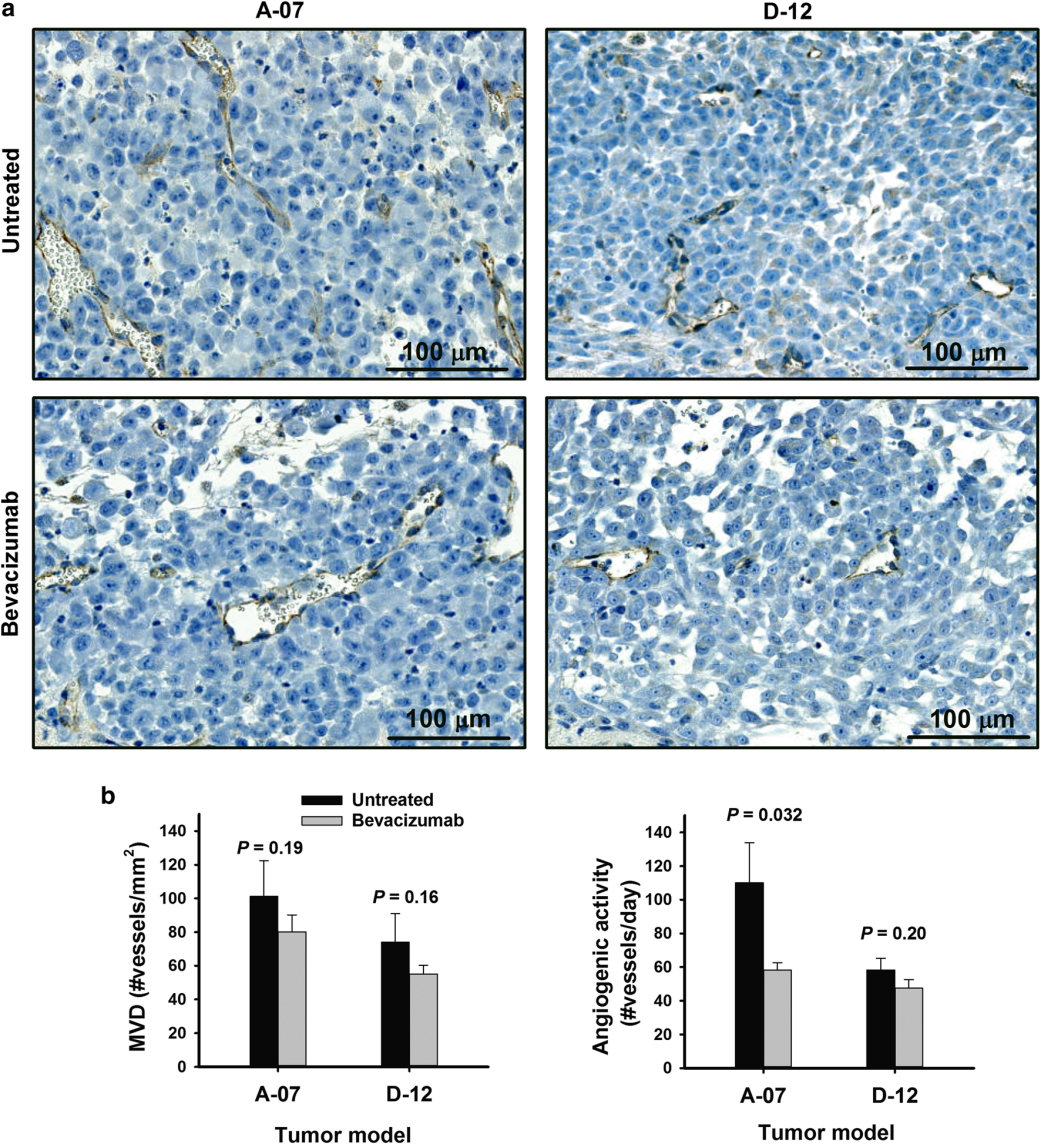
Microvascular density and angiogenic activity. **a** CD31-stained histological preparations of untreated and bevacizumab-treated meningeal A-07 and D-12 tumors illustrating that the tumors were vascularized by abnormal vessels. **b** Microvascular density (MVD) and angiogenic activity in untreated and bevacizumab-treated meningeal A-07 and D-12 tumors. Bevacizumab treatment did not reduce MVD in any of the models, whereas angiogenic activity was reduced in bevacizumab-treated A-07 tumors but not in bevacizumab-treated D-12 tumors. Columns: mean of 5 tumors. Bars: standard error. *P*-values: one-sided *t*-test

Meningeal A-07 and D-12 tumors showed substantial angiogenic activity. CD31-stained histological preparations revealed highly vascularized tumors with enlarged and irregular blood vessels in both models, regardless of whether the tumors were treated with bevacizumab or not (Fig. 2a). MVD did not differ between bevacizumab-treated and untreated tumors in any of the models [*P* = 0.19 (A-07) and *P* = 0.16 (D-12); Fig. 2b]. The angiogenic activity was lower in bevacizumab-treated than in untreated tumors in the A-07 (*P* = 0.032) but not in the D-12 (*P* = 0.20) model (Fig. 2b), implying that bevacizumab inhibited angiogenesis in meningeal A-07 tumors but not in meningeal D-12 tumors.

### Bevacizumab-treated meningeal A-07 tumors showed increased cerebral invasion

**Fig. 3:**
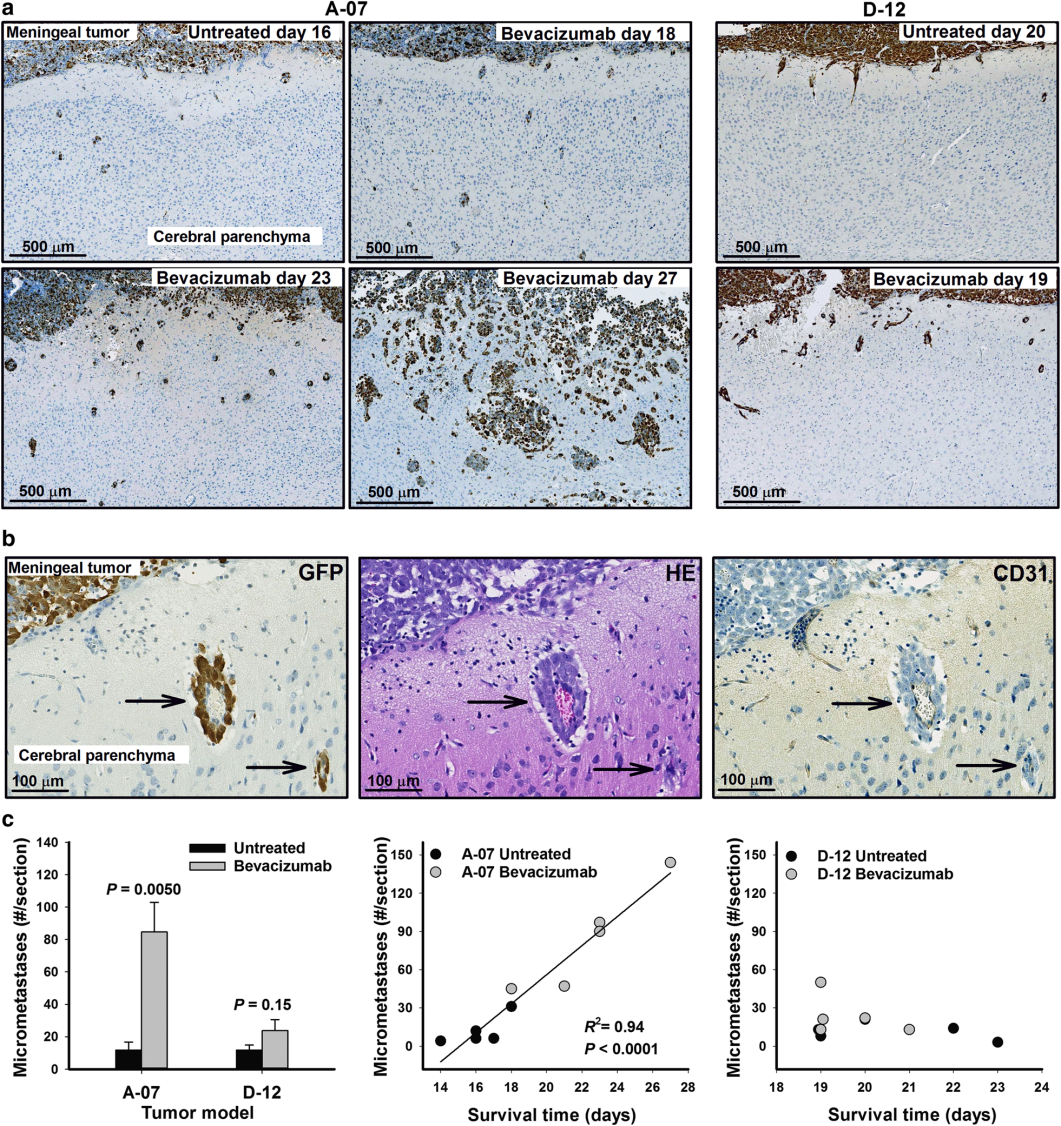
Invasion of meningeal tumors into the cerebrum. **a** GFP-stained histological preparations of the meningeal-cerebral border in untreated and bevacizumab-treated mice showing that A-07 and D-12 meningeal tumors invaded the cerebral parenchyma and developed micrometastases. **b** Adjacent GFP-stained, HE-stained, and CD31-stained histological preparations showing two cerebral A-07 micrometastases (arrows) supplied by a central co-opted vessel. **c** The density of A-07 cerebral micrometastases was higher in bevacizumab-treated mice than in untreated mice and correlated with host survival time, whereas the density of D-12 cerebral micrometastases did not differ between untreated and bevacizumab-treated mice and was independent of host survival time. Columns: mean of 5 tumors. Bars: standard error. Points: individual mice. Curve: linear regression. *P*-values: two-sided *t*-test (left panel) or Spearman rank order test (middle panel)

Multiple colonies of GFP-positive tumor cells were observed within the cerebral parenchyma of mice with meningeal tumors of both models (Fig. 3a). Most of these micrometastases were located close to the meningeal border, and evaluation of GFP-stained, HE-stained, and CD31-stained adjacent histological preparations revealed that the micrometastases grew around and along cerebral blood vessels (Fig. 3b). These vessels had a normal morphology, suggesting vessel co-option rather than sprouting angiogenesis.

Examination of GFP-stained histological sections from mice with A-07 tumors revealed that the density of micrometastases was higher in bevacizumab-treated than in untreated mice, and furthermore, the density and size of the micrometastases increased with the survival time of the host mouse. This is illustrated in Fig. 3a, showing the meningeal border of an untreated mouse with a survival time of 16 days and bevacizumab-treated mice with survival times of 18, 23, and 27 days. Furthermore, the border between the meningeal tumor and the cerebral parenchyma appeared increasingly more diffuse with increasing mouse survival, suggesting continuous cerebral invasion of melanoma cells (Fig. 3a). Quantitative analysis confirmed that the density of micrometastases was higher in bevacizumab-treated than in untreated A-07 mice (*P* = 0.0050; Fig. 3c), and moreover, there was a strong positive correlation between the density of micrometastases and host survival time (*P* < 0.0001; Fig. 3c).

The morphological appearance of the micrometastases from D-12 meningeal tumors was similar to that of the A-07 micrometastases. However, the density of micrometastases was not significantly different in bevacizumab-treated and untreated D-12 mice (*P* = 0.15; Fig. 3c). Moreover, there was no correlation between the density of micrometastases and the survival time of the host mouse (*P* > 0.05; Fig. 3c).

### Angiogenesis was driven by different pathways in meningeal A-07 and D-12 tumors

**Fig. 4:**
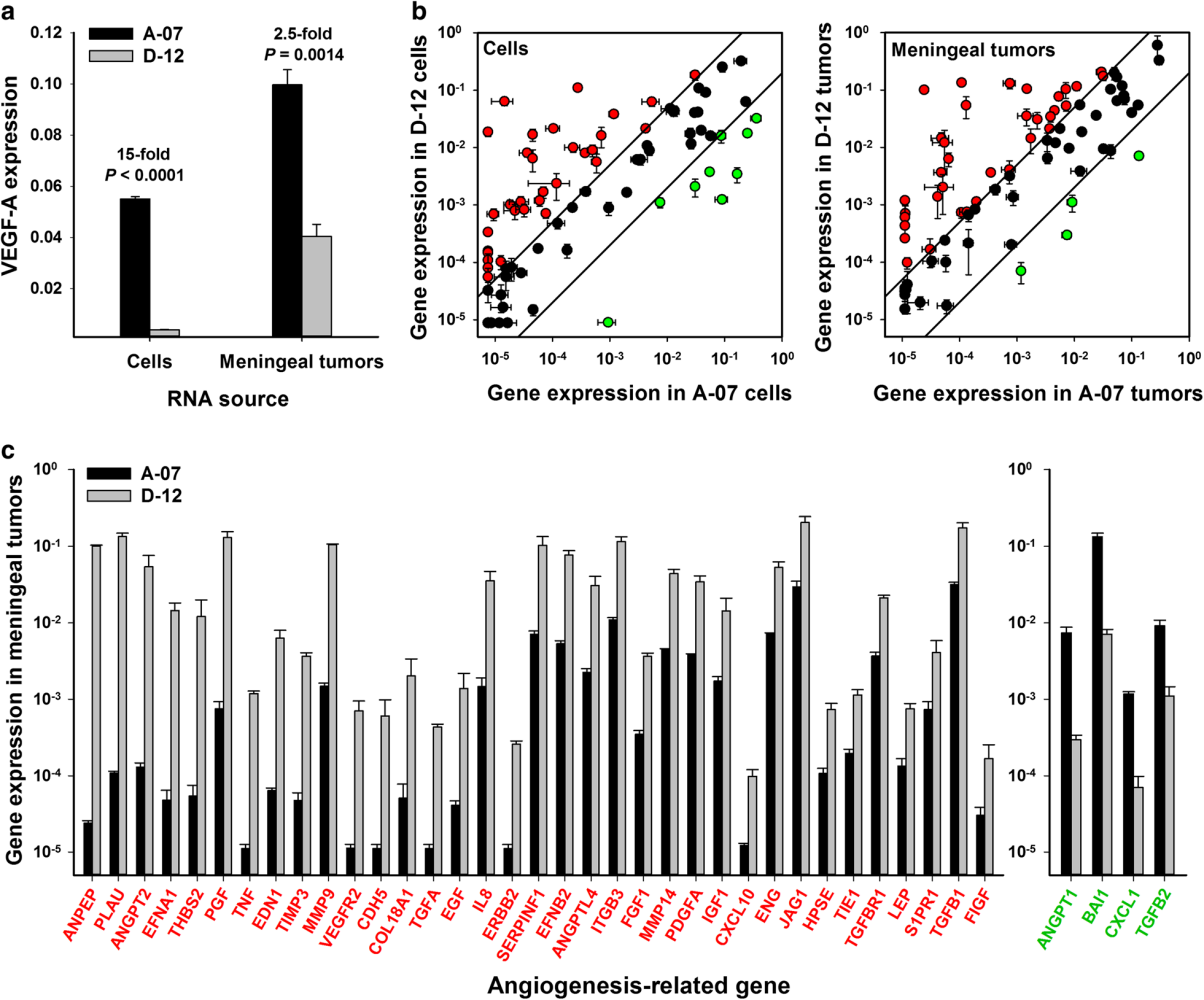
Expression of angiogenesis-related genes. **a** The expression of VEGF-A was higher in the A-07 model than in the D-12 model, both in cultured cells and meningeal tumors. **b** The expression of 84 angiogenesis-related genes in the D-12 model versus that in the A-07 model in cultured cells and meningeal tumors. The lines indicate a fivefold difference in expression level. Of the 84 genes, 38% (cells) and 42% (tumors) showed more than fivefold higher expression in the D-12 model than in the A-07 model (red symbols), whereas 11% (cells) and 5% (tumors) showed more than fivefold higher expression in the A-07 model than in the D-12 model (green symbols). **c** The expression of the specific genes showing expression levels differing by a factor of more than 5 between meningeal A-07 and D-12 tumors. Gene expression was measured with quantitative PCR and normalized to the expression of 5 housekeeping genes. Columns and points: mean of 3 cell cultures or tumors. Bars: standard error. *P*-values: two-sided *t*-test

To search for possible explanations of the differential responses to bevacizumab treatment of A-07 and D-12 tumors, the expression of angiogenesis-related genes in cultured cells and meningeal tumors was assessed by quantitative PCR. The expression of VEGF-A, the target of bevacizumab, was significantly higher in the A-07 model than in the D-12 model, both in cell cultures and meningeal tumors (*P* < 0.0001 and *P* = 0.0014, respectively; Fig. 4a). Of the 84 genes included in the PCR array, as much as 38% (cells) and 42% (tumors) showed more than fivefold higher expression in the D-12 model than in the A-07 model (Fig. 4b). On the other hand, only 11% (cells) and 5% (tumors) of the genes showed more than fivefold higher expression in the A-07 model than in the D-12 model (Fig. 4b). The expression of the specific genes showing expression levels differing by a factor of more than 5 between meningeal A-07 and D-12 tumors is presented in Fig. 4c. Interestingly, the genes encoding angiopoietin-2 (ANGPT2) and interleukin-8 (IL8), two well known drivers of angiogenesis in melanoma, showed substantially higher expression (416-fold and 24-fold, respectively) in D-12 tumors than in A-07 tumors.

### Bevacizumab-treated meningeal A-07 tumors showed increased expression of angiogenesis-related genes

**Fig. 5:**
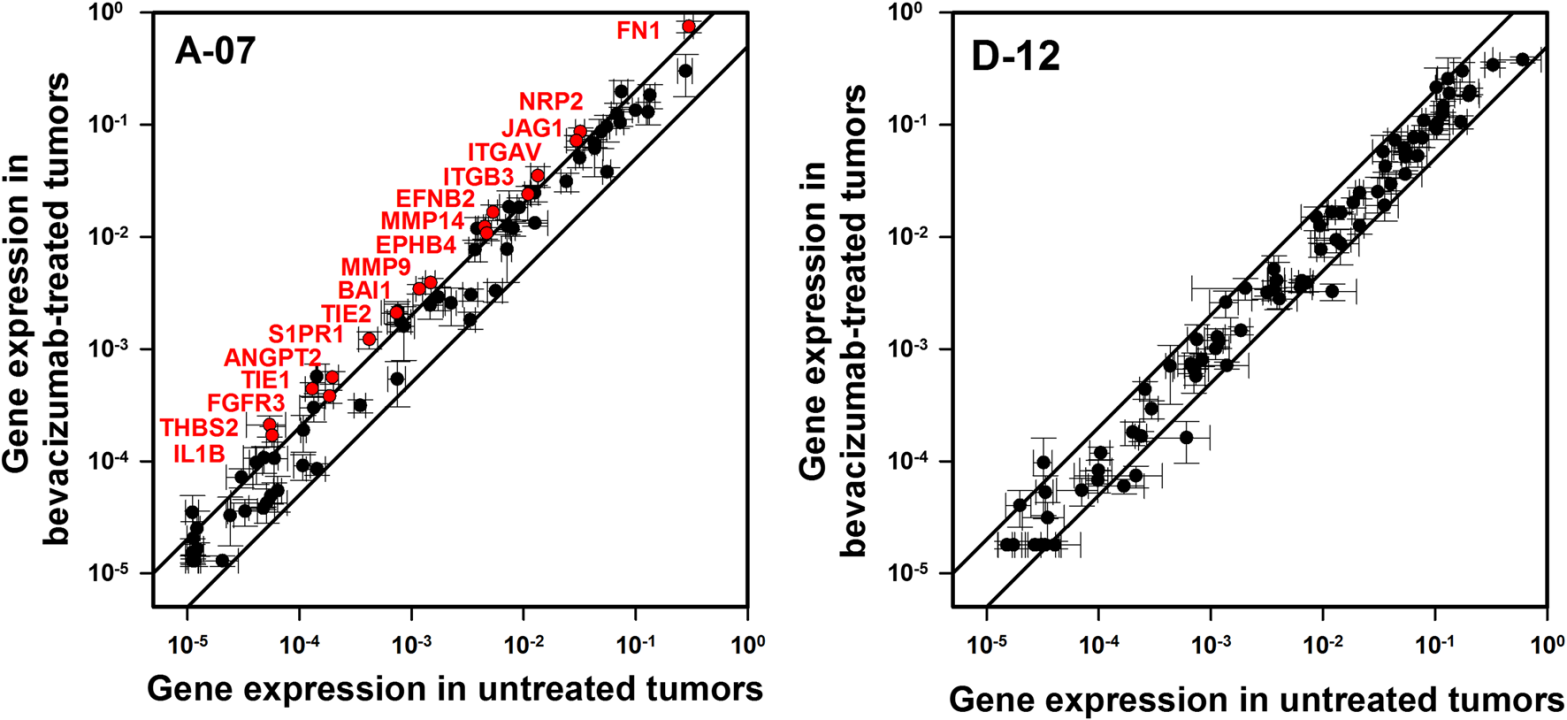
Changes in the expression of angiogenesis-related genes after bevacizumab treatment. The expression of 84 angiogenesis-related genes in bevacizumab-treated versus that in untreated meningeal A-07 and D-12 tumors. The lines indicate a twofold difference in expression level. Of the 84 genes, 17 (20%) showed a significant and more than twofold higher expression in bevacizumab-treated than in untreated A-07 tumors (red symbols), whereas none of the 84 genes showed expression levels that were significantly different in bevacizumab-treated and untreated D-12 tumors. Gene expression was measured with quantitative PCR and normalized to the expression of 5 housekeeping genes. Points: mean of 3 tumors. Bars: standard error

To investigate whether bevacizumab treatment induced changes in the angiogenic profile of meningeal tumors, tumor tissue from bevacizumab-treated and untreated mice was subjected to quantitative PCR. In A-07 tumors, 17 of the 84 genes (20%) showed a significant and more than twofold increase in expression after bevacizumab treatment (Fig. 5). These genes included ANGPT2 and its receptors TIE1 and TIE2, the matrix metalloproteases MMP9 and MMP14, and the genes encoding the two subunits of integrin αvβ3 (ITGAV and ITGB3). In contrast, the expression levels in bevacizumab-treated and untreated D-12 tumors were not significantly different for any of the genes included in the analysis (Fig. 5).

## Discussion

The present study showed that bevacizumab treatment prolonged the survival of mice bearing meningeal A-07 tumors, most likely because bevacizumab reduced the angiogenic activity and growth rate of the tumor tissue. These observations are in accordance with previous studies of intradermal A-07 tumors, having revealed a close link between angiogenic activity and tumor growth rate [29], reduced angiogenesis after treatment with an anti-VEGF-A antibody [34], and reduced growth rate and vessel density after treatment with sunitinib, a tyrosine kinase inhibitor targeting the VEGF receptors (VEGFR) [35]. Moreover, our A-07 data are in agreement with clinical data suggesting that meningeal melanoma lesions are susceptible to antiangiogenic therapy [8, 28], and with a preclinical study showing prolonged survival of mice with leptomeningeal metastases treated with the endogenous angiogenesis inhibitor angiostatin [24].

Although the survival of mice with meningeal A-07 tumors was prolonged after bevacizumab treatment, the treatment was not sufficient to block tumor-induced angiogenesis. We cannot exclude the possibility that the angiogenic activity of bevacizumab-treated meningeal tumors was a result of continued VEGF-A activity. Because bevacizumab is specific for human VEGF-A, any contribution from stromal-derived VEGF-A would not have been inhibited by bevacizumab [15]. Moreover, the bevacizumab dose may not have been sufficiently high to neutralize all tumor cell-derived VEGF-A. Low doses are however more clinically relevant in a preventive setting due to long treatment periods, and Ilhan-Mutlu et al. [21] have demonstrated prevention of brain metastasis formation in a lung adenocarcinoma xenograft model using a substantially lower dose of bevacizumab than that used in our study (2.5 versus 10 mg/kg).

Despite a possible contribution from residual VEGF-A activity, it is more likely that other angiogenic factors contributed to the sustained angiogenesis of bevacizumab-treated meningeal A-07 tumors. In addition to VEGF-A, the angiogenesis of intradermal A-07 tumors has been shown to depend on multiple angiogenic factors, including IL8, basic fibroblast growth factor, and platelet-derived endothelial cell growth factor [34]. The meningeal A-07 tumors expressed a large number of angiogenesis-related genes, including the genes encoding these factors. Furthermore, several angiogenesis-related genes, including ANGPT2 and its receptors TIE1 and TIE2, were up-regulated after bevacizumab-treatment. Next to the VEGF/VEGFR pathway, the ANGPT/TIE pathway is the most important angiogenic pathway in melanoma [14]. Thus, bevacizumab-treated meningeal tumors compensated for the loss of VEGF-A activity by up-regulating other angiogenesis-related genes and activating other angiogenic pathways. Consequently, combined inhibition of multiple angiogenic stimulators may be necessary to prevent angiogenesis in meningeal metastases even from VEGF-A-dependent melanomas like A-07.

In contrast to meningeal A-07 tumors, meningeal D-12 tumors did not show inhibited angiogenesis and growth rate after bevacizumab treatment. It is likely that this difference was a consequence of the differing angiogenic signature of the two melanoma models. Compared with A-07 tumors, D-12 tumors showed low expression of VEGF-A, but high expression of a large number of other angiogenic stimulators, including IL8, ANGPT2, urokinase plasminogen activator (PLAU), MMP9, and platelet-derived growth factor A (PDGFA). These factors have been recognized as important drivers of melanoma angiogenesis [14], and may have contributed to the bevacizumab-resistant angiogenesis of meningeal D-12 tumors. Interestingly, previous studies have revealed that IL8 and ANGPT2 play important roles in the angiogenesis and spontaneous pulmonary metastasis of intradermal D-12 tumors [34, 36].

Importantly, intradermal D-12 tumors have also been shown to be sensitive to anti-VEGF-A treatment, since tumor angiogenesis and the development of pulmonary metastases were reduced significantly in tumor-bearing mice treated with a neutralizing antibody against VEGF-A [34]. The present study taken together with that study suggests that the susceptibility of melanoma metastases to VEGF-A-targeting treatments may differ among metastatic sites. Consequently, efficient antiangiogenic treatment of melanoma patients with metastatic growth in more than a single organ may require the use of inhibitors of multiple angiogenic pathways.

Tumor cells of both melanoma models were seen to migrate from meningeal tumors into the cerebral parenchyma where they formed micrometastases. Whereas the growth of the meningeal metastases depended on sprouting angiogenesis, the growth of the cerebral metastases depended on vessel co-option. This difference in vascularization was probably a consequence of differences between meningeal and cerebral blood vessel in their susceptibility to angiogenic factors secreted by melanoma cells [27, 37].

The growth of meningeal A-07 metastases was inhibited by bevacizumab treatment but not the growth of cerebral A-07 metastases, probably because of the distinctly different mode of vascularization. Vessel co-option has also been seen previously to infer resistance to antiangiogenic therapy in preclinical models of melanoma brain metastases [22, 23]. Kienast et al. [23] used a cranial window chamber model to show that bevacizumab inhibited angiogenesis and induced dormancy of metastases established from lung carcinoma cells but had no effect on melanoma metastases showing a co-optive growth pattern. Moreover, Leenders et al. [22] established cerebral melanoma metastases in mice by intracarotid artery injection of tumor cells and showed that antiangiogenic therapy with a VEGF targeting agent resulted in sustained tumor progression via vessel co-option.

In the present study, bevacizumab appeared to promote cerebral invasion of meningeal A-07 cells and increase the development of micrometastases rather than inhibit metastatic growth in the cerebral parenchyma. The brains were removed for histological examination when the host mice were moribund, and the observed increase in cerebral invasion and metastatic growth was most likely a consequence of the increased survival time of bevacizumab-treated mice, allowing the micrometastases more time to develop in treated than in untreated mice. This suggestion is supported by the observation that the density of micrometastases correlated strongly with mouse survival time. Furthermore, the size of the micrometastases appeared to increase with the density of cerebral lesions.

However, there is evidence that antiangiogenic therapy can increase the invasive and metastatic potential of tumor cells [38, 39]. The bevacizumab-treated meningeal A-07 tumors showed increased expression of a large number of genes, including MMP9, MMP14, and those encoding the two subunits of integrin α_v_β_3_. MMPs are proteolytic enzymes that degrade some components of the extracellular matrix, whereas integrins mediate cell–cell and cell-extracellular matrix interactions, and both protein families are associated with cancer-cell invasion and metastasis [40, 41]. MMP9 has been shown to enhance the invasiveness of A-07 cells in vitro [42] and to facilitate the migration of leukocytes across the membrane separating pia mater from the cerebral cortex [43]. We can therefore not exclude that bevacizumab treatment increased the cerebral invasion of meningeal A-07 tumors by up-regulating genes promoting tumor cell migration, a possibility that warrants further investigations.

While the prognosis of patients diagnosed with melanoma brain metastases remains poor, the incidence is expected to increase both as a result of a general increase in melanoma incidence and as a result of prolonged patient survival due to improved control of extracranial disease [44]. Novel strategies for the treatment of melanoma patients with brain involvement are therefore highly warranted. A great advantage of antiangiogenic drugs such as bevacizumab is that they target endothelial cells and can exert their effect without crossing the blood–brain barrier [21]. However, despite the important role of angiogenesis in melanoma progression and metastasis, the data reported here suggest that the development of antiangiogenic strategies for the treatment of melanoma brain metastases may be highly challenging as the outcome of treatment may depend on the intracranial site of metastatic growth as well as the angiogenic signature of the melanoma cells and the extent of treatment-induced changes in this signature.

## Conclusions

Bevacizumab treatment inhibited tumor angiogenesis and prolonged tumor host survival in mice with meningeal A-07 tumors, but had no effect on the angiogenic activity of meningeal D-12 tumors. Meningeal A-07 tumors compensated for reduced VEGF-A-mediated angiogenic activity by up-regulating the expression of a large number of other angiogenesis-related genes, including genes governing the ANGPT/TIE pathway. Melanoma cells migrated from meningeal tumors into the cerebral parenchyma and formed micrometastases vascularized by vessel co-option, and in the A-07 model, bevacizumab-treated mice developed more and larger cerebral micrometastases than untreated mice. These discoveries suggest that antiangiogenic therapy may have the potential to inhibit the growth of meningeal melanoma metastases, but emphasize the need to target multiple angiogenic pathways and to individualize the treatment based on the angiogenic signature of the tumor tissue. Furthermore, antiangiogenic therapy cannot be expected to improve the outcome of meningeal melanoma metastases without being combined with therapeutic strategies for preventing tumor cell migration, vessel co-option, and metastatic growth in the cerebral parenchyma.

## Data Availability

The datasets used and analyzed during the current study are available from the corresponding author on reasonable request.

## Abbreviations

ACTB: β-actin
ANGPT2: angiopoietin-2
BALB/c: Bagg albino/c
B2M: β-2-microglobulin
BRAF: B-Raf proto-oncogene
GAPDH: glyceraldehyde-3-phosphate dehydrogenase
GFP: green fluorescence protein
HBSS: Hanks’ balanced salt solution
HE: hematoxylin and eosin
HEPES: 4-(2-hydroxyethyl)-1-piperazineethanesulfonic acid
HPRT1: hypoxanthine phosphoribosyltransferase-1
IL8: interleukin-8
ITGAV: integrin α-V
ITGB3: integrin β-3
MMP9 and 14: matrix metalloprotease 9 and 14
MRI: magnetic resonance imaging
MVD: microvascular density
PCR: polymerase chain reaction
RPLP0: ribosomal protein lateral stalk subunit P0
PLAU: urokinase plasminogen activator
PDGFA: platelet-derived growth factor-A
TIE1 and 2: tyrosine kinase with immunoglobulin-like and epidermal growth factor-like domains 1 and 2
VEGF-A: vascular endothelial growth factor-A
VEGFR: vascular endothelial growth factor receptor
